# Ecological, biological and social dimensions of dengue vector breeding in five urban settings of Latin America: a multi-country study

**DOI:** 10.1186/1471-2334-14-38

**Published:** 2014-01-21

**Authors:** Juliana Quintero, Helena Brochero, Pablo Manrique-Saide, Mario Barrera-Pérez, César Basso, Sonnia Romero, Andrea Caprara, Jane Cris De Lima Cunha, Efraín Beltrán - Ayala, Kendra Mitchell-Foster, Axel Kroeger, Johannnes Sommerfeld, Max Petzold

**Affiliations:** 1 Centro de Estudios e Investigación en Salud – CEIS, Fundación Santa Fe de Bogotá, Bogotá, Colombia; 2 Facultad de Agronomía, Sede Bogotà, Universidad Nacional de Colombia, Bogotá, Colombia; 3 Departamento de Zoología, Campus de Ciencias Biológicas y Agropecuarias, Universidad Autónoma de Yucatán, Mérida, México; 4 Departamento de Enfermedades Infecciosas y Transmitidas por Vectores, Centro de Investigaciones Regionales “Dr. Hideyo Noguchi”, Universidad Autónoma de Yucatán, Mérida, México; 5 Departamento de Protección Vegetal, Facultad de Agronomía, Universidad de la República, Montevideo, Uruguay; 6 Departamento de Antropología Social, Facultad de Humanidades y Ciencias de La Educación, Universidad de la Republica, Montevideo, Uruguay; 7 Centro de Ciências de Saúde, Universidad Esatdual do Ceará, Fortaleza, Brazil; 8 Departament of Public Health, Universidade Estadual do Ceará, Fortaleza, Brazil; 9 Departamento de Ciencias de la Salud, Universidad Técnica de Machala, Machala, Ecuador and Servicio Nacional de Control de Enfermedades Transmitidas por Vectores Artrópodos, Guayaquil, Ecuador; 10 Interdisciplinary Studies Graduate Program/Global Health Research Program, School of Population and Public Health, University of British Columbia, Vancouver, Canada; 11 Liverpool School of Tropical Medicine, Liverpool, and Programme for Research and Training in Tropical Diseases (TDR), World Health Organization, Geneva, Switzerland; 12 Special Programme for Research and Training in Tropical Diseases (TDR), World Health Organization, Geneva, Switzerland; 13 Centre for Applied Statistics, University of Göteberg, Göteberg, Sweden

**Keywords:** Dengue, *Aedes aegypti*, Vector breeding sites, Pupal indices, Urban settings, Ecobiosocial framework

## Abstract

**Background:**

Dengue is an increasingly important public health problem in most Latin American countries and more cost-effective ways of reducing dengue vector densities to prevent transmission are in demand by vector control programs. This multi-centre study attempted to identify key factors associated with vector breeding and development as a basis for improving targeted intervention strategies.

**Methods:**

In each of 5 participant cities in Mexico, Colombia, Ecuador, Brazil and Uruguay, 20 clusters were randomly selected by grid sampling to incorporate 100 contiguous households, non-residential private buildings (businesses) and public spaces. Standardized household surveys, cluster background surveys and entomological surveys specifically targeted to obtain pupal indices for *Aedes aegypti*, were conducted in the dry and wet seasons.

**Results:**

The study clusters included mainly urban low-middle class populations with satisfactory infrastructure and –except for Uruguay- favourable climatic conditions for dengue vector development. Household knowledge about dengue and “dengue mosquitoes” was widespread, mainly through mass media, but there was less awareness around interventions to reduce vector densities. Vector production (measured through pupal indices) was favoured when water containers were outdoor, uncovered, unused (even in Colombia and Ecuador where the large tanks used for household water storage and washing were predominantly productive) and –particularly during the dry season- rainwater filled. Larval infestation did not reflect productive container types. All productive container types, including those important in the dry season, were identified by pupal surveys executed during the rainy season.

**Conclusions:**

A number of findings are relevant for improving vector control: 1) there is a need for complementing larval surveys with occasional pupal surveys (to be conducted during the wet season) for identifying and subsequently targeting productive container types; 2) the need to raise public awareness about useful and effective interventions in productive container types specific to their area; and 3) the motivation for control services that-according to this and similar studies in Asia- dedicated, targeted vector management can make a difference in terms of reducing vector abundance.

## Background

Dengue is an emerging public health problem Latin America and the Caribbean; dengue incidence, as well as the frequency of outbreaks have dramatically increased during the last decade in the region [1]. According to World Health Organization (WHO), dengue transmission is currently reported in all Latin American countries, except for Uruguay [2-4]. In 2011, Brazil reported almost 71% (764,032) of all dengue cases for South Cone (807,191), followed by the Andean region (11% of cases, principally reported in Colombia, 33,207 cases, and Ecuador, 7659 cases, and by Mexico (6.3% of 67,918 cases) [5].

Dengue virus is transmitted by *Aedes aegypti* and occasionally by other species such as *Aedes albopictus*. Vector breeding sites are most commonly found in the intra- and peri-domestic environment, however, pre-imago stages have been found in public spaces, cemeteries, schools, hospitals, health centres and hotels [6,7]. Breeding sites of *Aedes aegypti* are closely related to macro- and micro-ecological factors that are determined by human behaviours - individual, collective and institutional - and their related social, economic and political contexts. Ecological, biological and social (i.e., “eco-bio-social”) variables are interdependent factors for dengue vector production with a direct impact, however complex, on dengue control measures and prevention [7-10]. Ecological factors refer to climate (rainfall, humidity, temperature etc.) and the natural and man-made ecological setting (including the urban, peri-urban and agricultural environment etc.). Biological factors relate to the behaviour of *Ae. aegypti*, and the transmission dynamics of dengue virus (vector population dynamics and feeding behaviour). Social factors incorporate a series of variables relating to health systems, including vector control and health services, and their political context (e.g. health sector reforms), public and private services such as sanitation and sewage, garbage collection and water supply as well as “macro-social” events such as demographic growth and urbanization, as well as community and household-based practices, knowledge and attitudes and how these are shaped by large-scale forces such as poverty, social inequality and community dynamics [11]. This broad eco-bio-social conceptual framework informed the present investigation of the ecosystem--specific (i.e. ecohealth) context in participant research sites. The research effort reported here is based on a longstanding research partnership between the Special Programme for Research and Training for Tropical Diseases (TDR) at the World Health Organization with the Ecosystems and Human Health (EcoHealth) programme of Canada’s International Development Research Centre (IDRC). Earlier, pilot research towards a comprehensive understanding of dengue vector development was conducted in Brazil [12] and in Colombia [13] and later on a comprehensive study in six Asian countries was conducted [10]. This programme lead to developing tools and strategies for community-focused partnership models for dengue vector management with a spatial perspective (neighbourhoods and their surrounding; public and private spaces) rather than the more traditional, however, restrictive household-based perspective [14]. The recognition of both private (intra- and peri-domestic) and public spaces, as well as the varied ecological characteristics of different kinds of urban neighbourhoods, has helped to cultivate a better understanding of vector dynamics and broaden the view for vector control interventions [15]. Clearly, the local transmission of dengue in Latin America are different from those in Asia [10-15]. Different socio-economic, including housing conditions, variable delivery mechanisms and quality of public services, local variability in water storage practices, different social structures and community dynamics, and vector control practices, both at the municipal as well as personal levels [16,17]. The study reported here is a multi-country research effort with a universal core protocol developed following a TDR/IDRC proposal development/study design/methods workshop on Innovative Community Based Ecosystem Management Interventions for Improved Chagas and Dengue Disease Prevention in Latin America and the Caribbean, held in Antigua, Guatemala in July 2009, and a Third Community of practice workshop held in Merida, Mexico, in August 2011.

The jointly developed protocol was applied in five Latin-American study sites in two phases. The purpose was 1) to explore in the first phase (which is the subject of this paper) the ecological, biological, and social (“eco-bio-social) factors that have contributed to the development of increased dengue mosquito populations in high-burden countries of Latin America 2) for comparative purposes, in a country where the vector is present but no dengue cases have yet been reported (Uruguay) and 3) to identify options for innovative community-based ecosystem management interventions to be designed, implemented and evaluated in phase 2, with active participation of all stakeholders involved, including communities, their governing structures (policy and decision makers etc.) and related services (water supply, waste management etc.).

## Methods

### Study sites and timeline

The study was conducted simultaneously in defined area clusters of urban sites of five countries (Mexico (Acapulco), Colombia (Girardot), Ecuador (Machala), Brazil (Fortaleza) and Uruguay (Salto) from November 2010 to August 2011. Table 1 provides an overview of the study sites. Four of the 5 study sites are located in dengue endemic areas with climatic conditions favourable for the continuous maintenance of *Aedes* vectors. The Uruguay site is the exception: it is adjacent to dengue endemic areas and is subtropical, sufficient survival time of dengue vectors to accommodate the incubation period required for viral dissemination and transmission is only possible for 5 months of the year due to climatological factors [18].

**Table 1:** Demographic, geographical and climatic characteristics of Latin America study sites

**Country**	**Mexico**	**Colombia**	**Ecuador**	**Brazil**	**Uruguay**
**Study sites**	Ciudad Renacimiento	Girardot	Machala	Fortaleza	Salto
**Total population for study site**	48460	132.456	281.500	2.447.409	123.000
**Mean 46 population per study area cluster (per season)** Dry – wet season	453 (370–542) 416 (313–526)	366 (261–454) 373 (215–479)	399 (364–449) 403 (357–438)	354 (202–454) 355 (202–462)	307 (221–418)
**Geographical position**	32º43′-14º32′ N 86º42′-118º22′ W	4°18′ 18″ N 74°48′ 06″ W	3.26°S 79.97° W	3°43′01″ S 38°32′35″ W	31º23′S, 57º58′
**Average annual temperature (°C)**	27.8 (Max 38.7; Min 16.2)	28.0 (Max 38.3; Min 23.2)	25.0 (Max 34; Min 18)	30.0 (Max 27; Min 23)	18.1 (Max 24.1; Min 12.5)
**Mean annual relative humidity %**	75	61,5	84	90	72
**Annual rainfall in 2011 (mm)**	1145	530	448	1378	1322
**Rainy season(s)**	May to October	March to April and October to November	November to April	February to May	Irregular; potential virus development only from mid November to April

### Area cluster definition and sampling

A sample of clusters was randomly selected for conducting all study surveys (household, entomological and cluster surveys) in each urban site. A cluster was defined as a geographical area that includes at least 100 private households, and incorporates the non-residential private buildings (businesses) and public spaces.

Public spaces in this study were defined as public streets or pathways, green spaces (parks), sports fields and paved courts, cemeteries, abandoned areas and dumping grounds, as well as public buildings like schools, day cares, hospitals or governmental offices, and religious buildings.

### Sample size calculation

The sample size was calculated as required for the cluster randomized intervention studies to be also conducted during phase II of this research project. It was based on a post-intervention cross-sectional comparison of the number of pupae per person in the intervention and control clusters using a two-level hierarchical model with clustering at the cluster level. The sample size reflected a desired power of 80% with the significance level set at 5%. The mean number of pupae per person in control and intervention clusters was assumed to be 3.0 and 0.3, respectively, based on previous studies [10,19]. For a negative binomial distribution with a dispersion coefficient of 0.02 and an intra-cluster coefficient of 0.05, 8.9 clusters with 100 households per cluster were needed per study arm [20], and the number was increased to 10 per study arm (i.e. 20 clusters for the study site). We assumed a negative binomial distribution to ensure a large enough sample, even if it was not clearly needed.

Following the later rationale, 20 study clusters were included in each site (In Brazil the household survey was limited to 10 clusters due to human resources constraints but the entomological surveys during the dry and wet season were done in all 20 clusters).

### Grid sampling of study clusters

**Figure 1:**
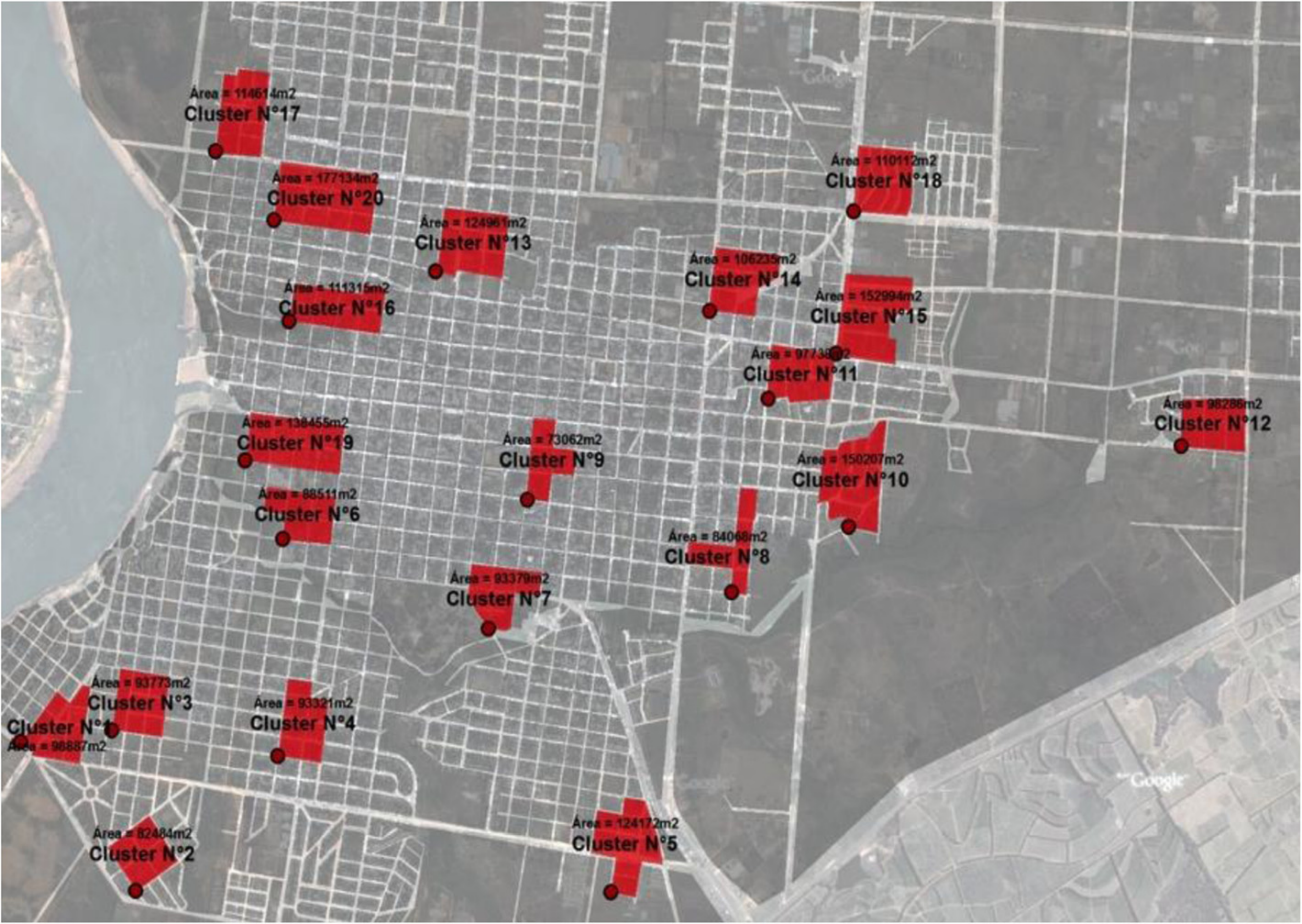
Grid sampling in Salto, Uruguay with cluster number, location and area.

A map of each study site was taken using Google Earth software [21,22]. A grid with 200 squares (or grid cells) was placed upon it (See example in Figure 1). The squares were numbered and 20 squares were randomly selected using simple random numbers (see sample size calculation). Selected clusters were a minimum of 500 meters (in one site 100 meters) apart as a measure to prevent cross-contamination of *Ae. aegypti* from one cluster to the next [23,24]. The left lower corner of each of the selected grids was identified and located geographically through geographic information systems (GIS) methods in the field. The closest street crossing to the physically located point was identified as the anchor of the cluster; one street representing the first vertical side of the square and the second street representing the first horizontal side of the cluster (square) on the GIS map. Proceeding from the anchor approximately 100 meters along the horizontal street (margin of the cluster), the next “vertically” oriented street to the left was established as the second vertical side to the cluster (parallel to the first vertical street) resulting in a 3-sided U shaped form. In order to close the U and complete the delineation of the cluster, 100 households (houses, flats, small business units) were recruited from within the U shaped area. Upon completion of household recruitment, the fourth side of the cluster is established beyond the property line of the final household and a map was drawn. If the square fell over a football ground or large park or any other open public space, then the next corner of two crossing streets was taken and the U was constructed to include these spaces.

### Data collection instruments

Experienced staff, either from the vector control services or biologists, administered the following three standardized research instruments. Quality control of the data was a routine part of field supervision.

### Cluster and household surveys

Two common instruments, a cluster characterization survey and a structured questionnaire for household interviews (both based on the lessons learned in the previous multicentre study in Asia [10]) were developed after the first meeting of Principal Investigators in Antigua, Guatemala, in July 2009. The characteristics of the study clusters included: size and geographical position of the cluster, socio- economic characteristics, type and overall quality of houses, characteristics of public spaces around the houses, basic infrastructure and public services as well as information about the ecological conditions around the clusters. In the household questionnaire questions were included on demographic characteristics, housing conditions (purpose of building, number of floors, construction material, protection of windows, characteristics of the peri-domestic area; water supply and storage, container management, toilets, waste disposal) and other environmental factors (trees or bushes around the house) as well as knowledge, attitudes and practices (KAP) about dengue and preventive practices both by the community and the government.

### Larval and pupal productivity surveys

Larval/pupae surveys were conducted in the dry and wet season according to standard operating procedures (SOP; [25]). Briefly, all the water holding containers of inspected premises were classified according to type, source of water, volume, location, the presence of vegetation, the presence of larvae control measures and the presence of a lid. Surveyors determined the presence or absence of larvae, and counted the total number of pupae in the following way: If the water containers had less than 20 L of water all pupae were counted. For containers with 20-100 L of water, all pupae were collected by comprehensive netting. For large containers, (>100 L) a sample of water was taken and a correction factor was applied (this was mainly the case in the Colombian site, see [25,26]). A sample of larvae was collected and identified to species in the laboratory. In addition, a sample of pupae (10%) from each container type was transported to the laboratory, reared to emergence, and the adults identified by species and sex.

### Data management and analysis

A data entry and management software was developed and managed by a central data management centre (DMC) at the Universidad del Valle, Guatemala. The web base software tool incorporated range and skip check and capability to facilitate standardized data collection from all study sites. Personnel from each study site had access to password protected data entry functions that allowed them to enter, submit and update data in real time (web site http://www.dengue-la.net). Site-specific data were sent to the DMC where the databases were checked for missing/excess values, cleaned, merged and finally sent on to the data analysis centre (DAC) at the University of Gothenburg, Sweden. Before entering into software all data gathered was double checked by field supervisors. Stata Version 12.1 (StataCorp LP, College station, TX, USA) was used for data analysis.

a) The statistical analysis was developed for container as one of the unit of analysis. Models for wet and for dry season were developed. Predictors of number of pupae per container were identified using a negative binomial regression as count data were being analyzed. Clustering of observations at study cluster level was assumed. The command nbreg with a clustered sandwich estimator in Stata 12.1 was used.

Potential important covariates were included in the regression models based on significant level and assumed dependencies. At cluster level entomological indices were calculated, then presented descriptively in relation to factors associated with vector productivity (estimated by pupal indices, [27]). No household level analysis was considered, there were very few individuals per hectare and often 0 pupaes.

### Ethical aspects

The research protocol was approved by the ERC (Ethical Review Committee) at World Health Organization (WHO) in Geneva and by local Institutional Review Boards (IRBs) in each respective participant country ( Mexico: Secretaria de Salud de Guerrero; Colombia: Fundación Santa Fe de Bogotá; Ecuador: University of British Columbia; Brazil: Universidad de Estadual do Cesará; Uruguay: Faculty of Medicine of Universidad de la República). All respondents signed a consent form before participating in the surveys.

## Results

### Characteristics of the study populations

A total of 9.213 householders in 90 clusters (20 in Mexico, Colombia, Ecuador and Uruguay and 10 in Brazil) were interviewed; each cluster had 100 households (in Brazil 125 households) and an average 385 inhabitants (3.85 persons per household, range 3.37 in Uruguay to 4.54 in Mexico). Respondents had an average of 8 to 9 years of schooling with a higher proportion of respondents with secondary education in Colombia and Uruguay and a considerable proportion without any school education in Mexico (15%) and Brazil (12.9%). The mean age was 42 to 48 years and 63.7% to 72.7% were women. The average tenure of permanent residence in recruited households was 18 to 23 years; only in the Colombian site a small proportion of families (4.4%) were weekend residents.

### Characteristics of the study clusters

The living conditions in the study areas are detailed in Table 2 and summarized in Table 3. Most clusters in Colombia, Ecuador and Uruguay were purely residential, while in Mexico and Brazil most sites were a mix of residential and commercial areas (predominantly small shops and restaurants). Most houses had only one floor and only a few neighborhoods in Uruguay, Colombia and Ecuador had multi-story buildings. The housing conditions were particularly good in Colombia and satisfactory in the majority of clusters in the other countries. According to the household interviews, most buildings were concrete (88.6% to 99.9%) and very few in Colombia and Mexico were made of wood (3.1% and 3.8% respectively).

**Table 2:** Living conditions in 20 clusters per study site according to household survey and cluster background survey

	**Mexico**	**Colombia**	**Ecuador**	**Brazil**	**Uruguay**
**Social class**	20/20 Lower middle	14/20 Lower middle	17/20 lower middle	6/10 lower middle	14/20 lower middle
Purpose of buildings*	18/20 residential & commercial	16/20 residential	14/20 residential	7/10 residential & commercial	14/20 residential
**Housing conditions**	18/20 satisfactory	17/20 good	15/20 satisfactory	8/10 satisfactory	15/20 satisfactory
Houses with one floor**	20/20	19/20	19/20	10/10	17/20
**Neighboorhoods with green areas**	4/20	18/20	10/20	0/10	19/20
**Neighborhoods with public recreation areas**	11/20	17/20	10/20	1/10	11/20
**Houses with glass windows**	23,1%	77,9%	61,20%	22,00%	94,90%
**Houses with backyards**	45,2%	82,2%	76,60%	74,10%	92,30%
**Indoor WC**	82.3%	74.9%	67.9%	95.5%	95.4%***
**Water storage (% of houses)**	99.0%	89.0%	89.0%	64.5%	11.2%
Stored water for drinking****	34.6%	10.4%	66.9%	13.7%	1.9%

*Remainder in Mexico and Brazil residential, in Colombia, Ecuador and Uruguay mixed (commercial /residential).

**Remainder are premises with multi-storey buildings.

***including septic tanks ****The remainder for washing or house cleaning.

**Table 3:** **Narrative description of the socio-economic characteristics of vector breeding (exact numbers and percentages in Table**2**)**

**Study sities**	**Characteristics of the study city**	**Characteristics of study clusters**
**México**	Acapulco is a major seaport in Guerrero State. Tourism is the main economic activity; most inhabitants are involved in commerce, touristic/hotel/restaurant services & transport. 34.6% of dengue cases in Guerrero state occurred in Acapulco (1556 of 4493). The study area “Ciudad Renacimiento” is on the north side of Acapulco built as a “social project” for people living on the hills. It is primarily residential, but also with schools, small businesses, markets, automobile & tyre repair shops. It is a high-risk area for dengue.	Mainly lower middle class residents in satisfactory housing conditions. Mixed commercial (restaurants, and shops) and residential neighborhoods. The one-floor houses had usually open windows (one quarter with glass), most had indoor flush toilets and half of them backyards. Water storage was frequent, mainly for washing and cleaning but also for drinking (35%). Green areas in the neighborhood and recreational areas were rare. Public infrastructure and waste collection was good.
**Colombia**	Girardot is a municipality in the department of Cundinamarca. It is the second most important city of the department, located along Magdalena river which makes it a central spot for commerce, communication and tourism. Due to its proximity to Bogota (129 km), particularly on weekends many visitors are in the city. Day temperatures vary between 25–30. The Department belongs to the areas with highest dengue endemicity in the country. Girardot reported 50% of all dengue cases in the department.	Mainly lower middle-class residents in good housing conditions. Mainly residential areas. Some multi-storey buildings. The one-floor buildings had usually glass windows, indoor flush toilets and more than 80% had back yards. Water storage was frequent, mainly for washing and cleaning. Recreational areas and green areas were frequent. Public infrastructure and waste collection was good.
**Ecuador**	Machala, the capital of El Oro Province, lies on the Pacific Coast, is situated within an agricultural region (banana, cacao, shrimp), intensive production has contaminated the environment and watershed with agrochemicals. The Greater Municipality of Machala is marked by patchy provision of adequate basic sanitary infrastructure (piped water, sewers and paved roads often lacking in peri-urban communities) and continued rapid, unplanned urbanization; 41.4% of the population lives below the poverty line. Peri-urban neighbourhoods continue to expand into “unauthorized” territory. Dengue is a major Public Health issue.	Mainly lower middle-class residents in satisfactory housing conditions. Mainly residential areas. Some multistory buildings. The one floor buildings often had indoor flush toilets (68%), glass windows (61%) and a back yard. Water storage was frequent, mainly for drinking (67%) but also for washing and cleaning. Half of the neighborhoods had green and recreational areas. Public infrastructure and waste collection was good some with poor access roads.
**Brazil**	Fortaleza is the Capital of Ceara State in Northeast Brazil. The city has a high-income concentration, with huge differences between the poorest and the richest. The city is located in a hot semi-arid region. The city is a high risk area for dengue.	Mainly lower middle-class residents in satisfactory housing conditions. Mixed commercial (restaurants, shops) and residential areas. The one-floor buildings had almost all indoor flush toilets but no glass windows (only 22%). Most had backyards. Recreational and green areas were very rare. Public infrastructure and waste collection was good.
**Uruguay**	Salto is located in the North-east of Uruguay on the Argentinian border. It has the characteristics of a “border-city” with heavy traffic of private vehicles, international passenger transport and truckload transportation from areas in which the vector is present and cases of dengue are reported.	Mainly lower middle-class residents in satisfactory housing conditions. Mainly residential areas; some multistory buildings. The onefloor buildings had almost all indoor flush toilets, glass windows and back yards. Green and recreational areas were frequent.

Glass windows were frequent in Uruguay (94.9% of houses), Colombia (77.9% of houses) and Ecuador (61.2%) and open windows were frequent in Brazil (78.0% of houses) and Mexico (73.7%). Most houses in Uruguay (94.9%), Colombia (82.2%), Ecuador (76.6%) and Brazil (74.1%) had back yards; fewer homes in the Mexico site had back yards (45.2%). Lower-middle class residents were the most frequent socio-economic group in all study clusters. However, in Colombia there was a considerable proportion of higher-middle class residents (probably due to the proximity of the capital city Bogotá) and conversely, in Ecuador a higher proportion of lower socio-economic strata (due to its location in an agricultural area; Table 3). The public infrastructure was generally good: the majority of clusters had asphalt streets (some clusters Ecuador with unpaved, poor quality roads and limited access to piped drinking water), electricity, piped drinking water and weekly removal of waste. Some of them had green and recreational areas, particularly in Colombia and Uruguay but much less in Brazil. Regarding the public spaces within the study clusters (not in the table). The most frequent public buildings were primary schools (64.4% of study clusters with at least one school), primary health care units (in 33.3% of the study clusters) and Christian churches in half of the study clusters with no major variation among countries. Cemeteries were not common within the clusters; only two in Colombia and one in Ecuador. Shopping malls were situated in some of the study clusters in Uruguay (5/20) and Ecuador (4/20) but only one in Colombia and one in Mexico. Tire capping facilities were particularly frequent in Uruguay (13/20 neighborhoods) and in Mexico (8/20).

### Water and sanitation

All study clusters had piped water. However, in Ecuador not all homes within the clusters had access to piped water. Moreover, water supply was irregular and not available through the public network on a daily basis; this is evident in that 20.2% of participant households in Ecuador obtained water from wells or from the river, this was almost negligible in the other study sites (less than 5% of households; Table 2). Households in all clusters stored water mainly to reduce the water bill rather than for human consumption; the water was predominantly used for washing or household cleaning (Table 2). In the 3 countries with strong water storage habits (Mexico, Colombia, Ecuador), small-volume water containers were emptied daily, the medium-sized containers weekly and the larger containers of more than 200 liters weekly or monthly. Larger water tanks were used for storage but (with the exception of Colombia) there were many other water holding containers –often rain filled- which were, based on the number and type of water containers encountered during the entomological surveys, more important for vector production (Table 4). In Mexico, Brazil and Uruguay 82.3% to 95.5% of houses had indoor flush toilets; in Colombia and Ecuador outdoor toilets were also frequent (24.5% and 23.4% respectively).

**Table 4:** Breeding places and infestation levels with immature dengue vectors in clusters

	**Mexico (n = 20)**		**Colombia (n = 20)**		**Ecuador (n = 20)**		**Brazil (n = 10)**		Uruguay**** (n = 20)	
**Season**	**Dry**	**Wet**	**Dry**	**Wet**	**Dry**	**Wet**	**Dry**	**Wet**	**Dry**	**Wet**
**Private water containers per cluster**	728	603	184	263	443	582	445	927	54	47
**% outdoor containers**	92.4	97.2	85.2	79.6	76.3	52.3	24.1	25.3	79.4	84.3
**Number of Public containers per cluster**	16	19	13	35	4	2	30	35	1	1
Most frequent container types*	Buckets, barrels, wash tanks		Wash tanks, barrels, buckets		Buckets, cans, wash tanks		Tires, barrels, buckets		Buckets, wash tanks	
Container types most frequently with larvae**	Tank 1.6% Barrel 1.3%	Can 19.1% Tire 15.0%	Can 44.1% Small cont. 34.3%	Tire 54.1% Tank 27.5%	Tank 21.7 Flower vase 20.8%	Tire 39.6% Tank 27.9%	Tire 7.1% Small cont. 1.7%	Nat. Prod 16.7% Tire 8.3%	Pot 60% Small Cont. 7.7%	Pot 70.6% Tire 55.5%
Most productive container types (% of all pupae)***	Bucket 34.5% Barrel3 0.6% Tank 23.1%	Small used 25.4% Bucket 21.0% Barrel 18.1% Cans 14.2%	Tank 71.2% Barrel 24.1%	Tank 72.5% Barrel 8.9% Tire 6.1%	Tank 47.9% Bucket 22.6%	Tank 35.5% Tire 15.9% Small Cont. 13.9% Cans 9.4%	Small cont. 50.9% Barrel 29.1%	Barrel 36.4% Cans 32.5% Bucket 8.0%	Barrel 65.3% Cans 34.7%	Cans 29.9% Others used 15.4% Bucket 13.9% Barrel 12.1%
**Number of pupae per cluster, rounded (with CIs)**	13 (6–20)	83 (53–112)	465 (270–661)	390 (293–488)	146 (97–195)	576 (419–734)	6 (0.6- 10.4)	54 (25–82)	4 (0–7.6)	20 (8–32)
**PPI (CIs)**	0.03 (0.01- 0.05)	0.2 (0.14- 0.26)	1.24 (0.73- 1.75)	1.03 (0.81- 1.25)	0.37 (0.25- 0.49)	1.42 (1.02- 1.82)	0.01 (0.00- 0.03)	0.15 (0.07- 0.23)	0.01 (0.00- 0.03)	0.07 (0.03- 0.11)
**PPH (CIs)**	2.4 (1.24- 3.64)	18.1 (12.8- 23.4)	296.1 (82.8- 510.0)	213.3 (103–323.7	35.0 (12.7- 57.2)	150.2 (68.1- 232.3)	1.8 (0.27-3.37)	29.7 (9.1- 50.3)	0.32 (0.00- 0.66)	1.7 (0.76-2.61)
**BI (CIs)**	5.5 (3.5- 7.3)	29.2 (23.6- 34.8)	29.2 (24.5- 33.8)	39.8 (33.5- 46.0)	32.9 (28.0- 37.8)	57.9 (48.6- 67.2)	3.3 (1.7- 4.8)	9.6 (5.9- 13.3)	0.7 (0.27- 1.06)	6.2 (4.0-8.5)

*The same water tanks in the dry and wet season, but rank order has changed in some cases.

**% of infested containers (of specific type) from all containers of that type; “small containers” were all un-used . ***% of all pupae encountered **** Uruguay has an irregular distribution of rainfall during the year; dry season corresponds to November until first two weeks in December and wet season to April until the first two weeks of May.

### Household knowledge and practices regarding dengue vector

Overall, the vast majority of respondents had heard about dengue and perceived the disease to be a problem (>95% of respondents). The most important sources of information were newspapers, radio, television and community health centres. Most respondents knew that dengue is transmitted by mosquitoes (80.1% to 93% in Colombia, Ecuador, Mexico but less in Brazil and Uruguay, 64.6% and 63.1% respectively) but only a minority was aware where mosquitoes lay their eggs (35.3% to 41.5%) with the exception of Mexico (74.0% were aware). About half of the respondents in Mexico, Colombia and Ecuador had seen larvae in their water (52.1% to 59.1%), but in Brazil only a few (32.3%) or in Uruguay almost none (8.8%). This is consistent with the different levels of entomological indices shown in Table 4: high awareness of larvae in sites with high vector densities, low awareness in sites with low vector densities. Personal protective measures against mosquitoes included the following (most frequently mentioned measures):

Mexico: garbage clean-up, larviciding, sticky tape.

Colombia: spraying of insecticides; garbage clean-up.

Ecuador: sticky tape, garbage clean-up, spraying of insecticides, eliminating plant or weed growth around the home.

Brazil: sticky tape, spraying of insecticides.

Uruguay: sticky tape, spraying of insecticides, repellents.

“To cover water containers” was only mentioned by a quarter of interviewees and “biological control” only by a minority in Colombia (16.1%).

### Current vector control by government and communities and expected actions from the government

The current vector control activities by the government vary among sites (Table 5). Mexico had the most continuous services with ultra low volume (ULV) space spraying (“fogging”) and larviciding as the predominant activities. Brazil was the second best-served site by vector control services with control agents doing garbage collection and larviciding as the most frequently mentioned activity followed by outdoor fogging. In Colombia, Ecuador and Uruguay the vector control services were irregular with outdoor fogging as the most frequently mentioned activity. Educational activities were rarely mentioned in all sites (range 32.8% in Brazil down to 13.7% in Uruguay).

**Table 5:** Current vector control activities by the government (n = number of respondents)

**Activities by vector control staff**	**Mexico (n = 2000)**	**Colombia (n = 1994)**	**Ecuador (n = 2000)**	**Brazil (n = 1251)**	**Uruguay (n = 1968)**
**Visit by control staff in last 6 months (%)**	96.5	31.5	27.5	92.16	29.8
**Inspect water (%)**	33.2	8.9	9.0	1.1	37.7
**Larviciding (%)**	72.5	24.9	11.7	5.0	0.25
**Indoor fogging (%)**	27.3	7.0	2.0	3.1	1.3
**Outdoor fogging (UVL) (%)**	87.3	67.1	67.2	34.8	57.0
**Health education (%)**	6.5	21.4	15.6	32.8	13.7
**Supply lids, recommend fish, cut plants**	Negligible	Negligible	Negligible	Negligible	Negligible

For all sites, respondants’ expectations regarding governmental vector control, reflected current government practices: ULV fogging was the most frequently mentioned expected measure, with the exception of Brazil where only 17.7% of respondents mentioned it.

The predominant response regarding community practices for the elimination of dengue mosquitoes in all sites was “nothing”: Brazil and Colombia had 95% non-participation, Uruguay had 84.1% non-participation, Mexico had 81.3% and Ecuador 77.4%. In Ecuador, 24.6% of respondents mentioned community clean-up efforts organized by neighbourhood councils in collaboration with the Municipal Government.

### Vector breeding and productive containers in the dry and wet season

Water filled containers were more frequent in the wet season compared to the dry season (Colombia, Ecuador and Brazil). Mexico was the exception with more containers for water storage (i.e. buckets, barrels and tanks) present during the dry season (Table 4). In Uruguay there was no consistent pattern as there was no marked wet and dry period. Most water containers were outdoors with the exception of Brazil where the water was mainly stored indoors (Table 4). In most sites there was only a negligible difference (< 5%) in the proportion of outdoor containers between the dry and wet seasons, with the exception of Ecuador where there was an increase of 24% in the number of outdoor water storage containers in the dry season). There were very few water containers in public spaces compared to private houses (Table 4). The most frequent container types were nearly uniform in the five study sites both in the dry and wet season: buckets, barrels and cement tanks for washing clothes; discarded tires were also frequent in Brazil. The average volume of water containers was fairly similar in 4 sites (range 100 L to 327 L) but was much higher in the Colombian site (1162 L, not in the table).

Nearlly all pupae eclosed from field samples (10% of total) in the laboratory were *Aedes aegypti* mosquitoes: 100% in Mexico, 100% in Colombia, 85% in Ecuador, 100% in Brazil and 87.0% in Uruguay. No *Aedes albopictus* were detected.

The container types most frequently infested with immature vectors (Ae. aegypti larvae and/or pupae) were tires, pots and cans. In contrast, the container types producing most *Ae. aegypti* (using as a proxy measure the number of *Aedes* pupae), were mostly the large container types like rectangular wash tanks and barrels; however, in Mexico, Brazil and Uruguay, buckets, cans and small discarded containers were also important producers of pupae (Table 4). In Colombia the large cement tanks alone produced more than 70% of *Aedes* pupae during the dry and wet season. In other study sites, two to four different container types produced more than 70% of *Aedes* pupae during the rainy season (Mexico, Ecuador, Uruguay; Table 4).

Comparing the study sites, Colombia and Ecuador had the highest vector indices (PPI in the wet season 1.03 and 1.42 respectively, Table 4). The entomological indices, particularly pupal indices (PPI and PPH) were considerably higher in the wet season compared to the dry season (PPI was 3.8 to 15 times higher during the wet season, Table 4). In Colombia the pupal indices showed no significant difference between the dry and wet season, likely due to the unusual rainfall during the dry season of 2010.

### Risk factors for vector breeding

The regression analysis of risk factors for vector production (pupae per container, Table 6) showed that outdoor location of water containers, non-use of the water in the container for more than a week, rainwater fill and uncovered of containers were significantly associated with higher vector production in the dry season.

**Table 6:** **Container characteristics associated **^**a **^**with the number of pupae per container identified as risk factors for dengue vector breeding during wet and dry season in private and public premises (outdoor and indoor)**

	**Dry season**			**Wet season**		
**Container**	**IRR**^**b**^	**95% CI**	**P-value**	**IRR**^**b**^	**95% CI**	**P-value**
**Not under shrubbery**	Reference			Reference		
Fully or partially under shrubbery	0.81	0.43-1.50	0.497	0.96	0.61- 1.49	0.862
**Used during past 7 days**	Reference			Reference		
Not used during past 7 days	3.48	1.71-7.05	0.001	2.44	1.60- 3.72	<0.001
**Fully covered**	Reference			Reference		
Partially covered	0.90	0.39-2.05	0.807	6.22	2.98- 12.96	<0.001
Not covered	15.10	7.70- 29.58	<0.001	9.88	5.67- 17.19	<0.001
**Localization indoor**	Reference			Reference		
Outdoor	3.87	2.03-7.36	<0.001	1.39	0.94- 2.04	0.092
**Faucet water**	Reference			Reference		
Rain water	3.42	1.14- 10.26	0.028	1.28	0.79- 2.06	0.315
**Filled with rain water and tap water**	5.20	2.21- 12.21	<0.001	1.24	0.62- 2.50	0.538

^a^Results of negative binomial regression with clustering at the study cluster level.

^b^Incidence rate ratio. Example: the expected pupal count for containers not used in the past 7 days is 3.48 times higher than that for containers used in the past 7 days.

During the wet season a lower significance level was observed for “non-use during past seven days” and the “rain fill” of containers was less important for pupal production. This may be due to the fact that intense rainfall in the wet season causes localized flooding and flushing of smaller containers, effectively washing away immature mosquitoes from breeding sites. Vegetation above the water containers did not significantly lead to increased vector breeding.

## Discussion

### Vector production in highly endemic areas

The urban settings of our study were quite well developed in terms of infrastructure (electricity, public water supply, paved streets), housing conditions (concrete constructions, sanitary facilities) and socio-economic status of the inhabitants (lower to upper middle class as assessed by interviewers). The populations were well informed about dengue and knew the essentials about the vectors. Domestic water management, however, was problematic: in all dengue endemic areas studied (Mexico, Colombia, Ecuador, Brazil) people routinely store water, mainly for washing and cleaning purposes. In Ecuador water is also stored for drinking. However, large water storage containers (>200 L) in Colombia (and to a certain extent in Ecuador) produced most of the Aedes aegypti mosquitoes, likely because of infrequent cleaning of the tanks.

In the Mexico, Brazil and Uruguay study sites, smaller and generally un-used containers like barrels, cans and pots were more important for vector production.

The regression analysis identified outdoor containers (especially during the dry season), uncovered containers and un-used containers as the main sources of vector production overall. Additionally, during the dry season rainwater-filled containers were important producers of dengue vectors. These findings are consistent with studies in Asia [10,28,29]. However, in the Latin American sites, as opposed to the Asian sites, water containers shaded by vegetation showed no significant increase in vector productivity compared to non-shaded containers.

This may be due to the nature of the vegetation itself; in Asia shrubbery and lower-lying plants were common shade agents, whereas shade in the Latin American study sites was provided by tall trees which would not provide the same microenvironmental or microclimatic conditions (including shade) for ovipositing mosquitoes.

The highest proportion of water containers infested with immature stages of *Ae. aegypti* (larvae and/or pupae) were un-used tires, small pots and cans (Table 4).

However, the major production of dengue vectors took place in other container types, mostly in large tanks or barrels (Table 4). This confirms a number of studies with similar results [10,28,30-33] underlining the need for complementing the routine “larval surveys” with occasional pupal productivity surveys [25].

### Vector production in low endemicity and at-risk areas

Uruguay represented a special case in our multi-centre study. The city of Salto is located at the edge of a dengue endemic area with no reported dengue cases but with documented presence of the dengue vector *Ae. aegypti*[2]. The climate in Salto is such that vectors survive long enough to accommodate the viral incubation period for only 5 months of the year, local transmission is biologically possible only during this period. Dengue herd immunity in the urban population of Salto can be considered to be close to zero as there has been no reported virus transmission in recent years. Therefore, according to the computer models by Focks et al. [34], the relatively low pupal density of PI = 0.07 at the end of the potential transmission season may be sufficient for dengue outbreaks in this susceptible population in the absence of a considerable rise of temperature.

Comparing Asian and Latin American study sites In the Asian study sites in India, Indonesia, Myanmar, Philippines, Sri Lanka, Thailand [10,28] there were higher human population densities (roughly 5 persons per household, only Thailand with 3.4, compared to the average of 3.8 persons per household in Latin America study sites), higher proportion of households were of lower socio-economic, poorer water and sanitation conditions (houses frequently without piped water and indoor toilets) and frequent tire capping facilities, but also with reasonable infrastructure (electricity, paved streets, regular solid waste collection).

In both regions people were well informed about dengue, but there was less knowledge about dengue vectors in the Asian sites.

The types of containers to store water were similar in both regions, with more frequent flower vases and ritual flower bowls in the Asian sites. The average number of water containers per study cluster was higher in Asia than in Latin America: 461 containers in Asia versus 127 containers in Latin America during the dry season and 508 containers in Asia versus 225 containers in Latin America during the wet season. However, the containers in Latin America were more productive for the development of dengue vectors: the number of pupae per cluster were in Asia and Latin America 82 and 127 respectively during the dry season and 130 and 225 respectively during the wet season. Likewise the PPI values in Asia were lower than those in Latin America (0.27 versus 0.57 during the wet season) in part due to higher population densities in the Asian sites. There were sites with high pupal production in Asia (Myanmar and Indonesia) and in Latin America (Ecuador and Colombia) and others with low pupal production (Asia: Thailand and Sri Lanka, Latin America: Uruguay and Brazil). Low vector densities in Brazil, Thailand and Sri Lanka were likely due to strong vector control services; additionally in Uruguay the climatic conditions were unfavourable to year round-vector survival. In both regions the identification of productive container types though pupal productivity surveys should be done during the wet season in order to identify all important container types; during the dry season container types that are important during the wet season cannot always be identified.

## Conclusions

In conclusion, our study has identified a number of action points to be taken into account to streamline vector control services and to increase program impact on vector indices:

1. Comprehensive and systematic eco-bio-social assessments of the local setting of vector breeding are a useful step in defining community-based and ecosystem-relevant vector control strategies.

2. Specifically, “larval surveys” used since the 1940’s in dengue vector surveillance are useful to establish presence or absence of dengue vectors in the community but should be complemented by occasional pupal productivity surveys [25,35,36] to identify productive container types for “targeted interventions”.

3. Interventions targeting productive container types have been shown to be more cost-effective than generalized breeding site reduction campaigns [7,37]. Targeted interventions are particularly effective in areas where only one or two container types produce most Aedes mosquitoes (in our study Colombia and Brazil, Table 4); targeted interventions may still offer increased benefit even if there are three or four productive container types for dengue vectors (Mexico and Ecuador in our study).

4. Pupal productivity surveys should be conducted during the wet season in order to identify all potentially productive containers (Table 4; see also Khin Thet Wai et al. 2012 [28]). Targeted interventions may influence vector breeding patterns over time, hence the pupal productivity survey should be repeated after a determined interval to establish newly important or alternative vector breeding sites.

5. High-risk container types for dengue vector development are outdoor, rarely used, uncovered, usually rainwater-filled and (particularly in Asia) shaded by shrubbery (see also Morrison et al. 2005 [29]).

6. Even in low endemicity areas or dengue-free but at-risk areas (Uruguay in our study) vector services should be aware of the dengue threat and keep the number of productive containers at a minimum, particularly during the possible transmission window.

7. Pupal production in non-residential areas (“public spaces”) is highly variable between sites. While in our and other study areas [8] public spaces contributed very little to increase the vector population due to the few water containers encountered in those areas, other pupal productivity surveys on cemeteries [7,38] or in and around public buildings [6] found pupal production to be as high in those places as in residential sites suggesting that pupal surveys should include non-residential spaces and buildings.

8. Well-organized vector control services including regular solid waste collection seem to make a difference regarding the reduction of vector abundance as seen in both Asian and Latin American sites [39-41].

## Abbreviations

TDR: Special programme for research and training for tropical diseases; WHO: World Health Organization; EcoHealth: Ecosystems and human health; IDRC: Human health programme of Canada’s International Development Research Centre; PPI: Pupae per person index; PPH: Pupae per hectare index; GIS: Geographic information systems; KAP: Knowledge attitudes and practices; SOP: Standard operating procedures; DMC: Central data management centre; DAC: Data analysis centre; ERC: Ethical Review Committee; IRB: Institutional Review Boards; LV: Ultra low volume.

## Competing interests

The authors declare that they have no competing interests.

## Authors’ contributions

JQ, PM, CB, AC and KF codirected the study and were responsible for overall project management in their respective country. Co- authors HB, SR, MB, JDL and EB were actively involved as members of team contributing to fieldwork, analysis and interpretation of data. MP participated in the initiall conception, design of the study and analysis of data in collaboration with AK and JS. JS led the critical revision of the manuscript. AK, PM and JQ prepared the first draft of this manuscript in collaboration with co-author CB and KF. All authors helped to write, revise and approve the final version of the manuscript. All authors read and approved the final manuscript.

## Pre-publication history

The pre-publication history for this paper can be accessed here:

http://www.biomedcentral.com/1471-2334/14/38/prepub
